# MetaFunc: taxonomic and functional analyses of high throughput sequencing for microbiomes

**DOI:** 10.1017/gmb.2022.12

**Published:** 2023-01-12

**Authors:** Arielle Kae Sulit, Tyler Kolisnik, Frank Antony Frizelle, Rachel Purcell, Sebastian Schmeier

**Affiliations:** 1 Department of Surgery, University of Otago, Christchurch, New Zealand; 2 School of Natural Sciences, Massey University, Auckland, New Zealand

**Keywords:** Metatranscriptomics, microbiome, functional annotation, host correlation

## Abstract

The identification of functional processes taking place in microbiome communities augment traditional microbiome taxonomic studies, giving a more complete picture of interactions taking place within the community. While there are applications that perform functional annotation on metagenomes or metatranscriptomes, very few of these are able to link taxonomic identity to function or are limited by their input types or databases used. Here we present MetaFunc, a workflow which takes RNA sequences as input reads, and from these (1) identifies species present in the microbiome sample and (2) provides gene ontology annotations associated with the species identified. In addition, MetaFunc allows for host gene analysis, mapping the reads to a host genome, and separating these reads, prior to microbiome analyses. Differential abundance analysis for microbe taxonomies, and differential gene expression analysis and gene set enrichment analysis may then be carried out through the pipeline. A final correlation analysis between microbial species and host genes can also be performed. Finally, MetaFunc builds an R shiny application that allows users to view and interact with the microbiome results. In this paper, we showed how MetaFunc can be applied to metatranscriptomic datasets of colorectal cancer.

## Background

Metagenomic or metatranscriptomic studies of microbiome communities allow for characterisation of functional contributions as well as taxonomic load, by allowing the identification and quantification of genes possibly contributed by the microbial community. The ability to identify functional processes from the microbiome gives a more complete picture of microbe–microbe and/or microbe–host interactions that drive community dynamics (Langille, 2018).

There are existing bioinformatics programmes (Nayfach et al., 2015; Sharma et al., 2015; Silva et al., 2016) that perform functional annotation on metagenomes and metatranscriptomes, but most of these are unable to link taxonomies (the microbes under study) to their respective functional processes. Existing packages with this capacity include PICRUSt and PICRUSt2 (Douglas et al., 2019; Langille et al., 2013), and HUMAnN2 (Franzosa et al., 2018). PICRUSt and PICRUSt2 predict metagenome function by inferring genes present in OTUs based on their phylogenetic similarities to other OTUs with known gene content (Douglas et al., 2019; Langille et al., 2013). However, they do not directly measure the genes involved, but rather rely on 16S gene marker sequences, which, being highly conserved, are useful for the identification of bacterial genera (Bashiardes et al., 2016; Ternes et al., 2020) and are not present in other microbes aside from Bacteria and Archaea (Ye et al., 2019). Thus 16S based taxonomic identification, and subsequent functional predictions, may be unsuitable for species-level identification, and for recognising other microbes aside from Bacteria and Archaea. HUMAnN2’s taxonomic profiling, meanwhile, is reliant on MetaPhlAn2 (Segata et al., 2012; Truong et al., 2015), which uses clade-specific marker genes from reference genomes. Benchmarking efforts by Ye et al. (2019) highlight the limitations of using the MetaPhlAn2 package, and therefore HUMAnN2, which results in relatively lower precision and recall in its classification.

To augment such meta-omic studies, we present here a simple, straight-forward pipeline named MetaFunc, a snakemake workflow (Köster and Rahmann, 2012) that maps function to a microbiome (and optionally host) sample, using RNA sequences as input. MetaFunc uses Kaiju (Menzel et al., 2016) as its main taxonomic classifier. Kaiju uses protein translations of input reads to generate taxonomic profiles. By generating protein-based classifications using metatranscriptomic reads, MetaFunc identifies microbes based on their gene expression, allowing more focus on the functional contributions of microbes. MetaFunc then uses protein accession numbers from Kaiju results to obtain the set of gene ontology (GO) terms associated with the microbiome community. Furthermore, Kaiju outputs provide a direct protein – taxonomy ID relationship that makes it possible for MetaFunc to establish which organisms are contributing to the functional GO terms. MetaFunc also has options for pre-processing of reads before running Kaiju: trimming of input reads with fastp (Chen et al., 2018) can be performed in addition to pre-mapping to a host genome (eg. human) using STAR (Dobin et al., 2013). The unmapped reads following STAR processing are the input used by MetaFunc for microbe identification, while host gene expression information can be obtained from STAR-mapped reads. Thus, MetaFunc allows simultaneous investigation of host and microbe community active functional processes, as well as active host genes and microbes.

## Protocol

### Workflow

**Figure 1:**
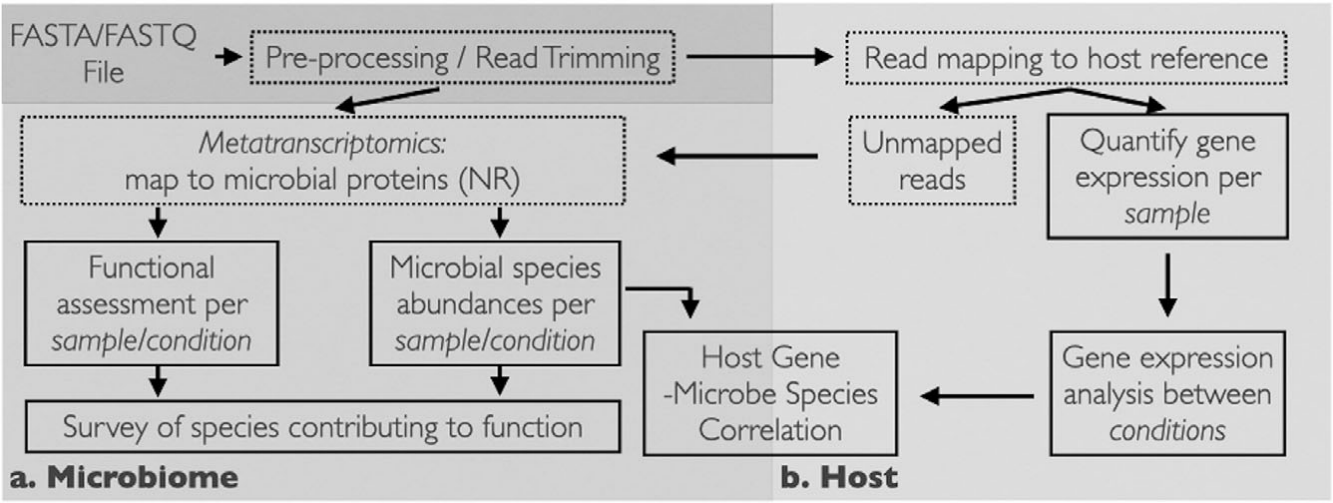
**MetaFunc Workflow.** The workflow uses FASTQ or FASTA as input and processes reads through the microbiome pipeline to give microbial abundance and function **(a)** and/or host gene analysis **(b)** which will first map reads to a host before sending unmapped reads to the microbiome pipeline. Applying host read analysis will give gene expression analysis results as well as host gene-microbial species correlation. Solid boxes indicate steps with an output while dotted boxes indicate intermediate steps in the pipeline. **NR**: NCBI Blast *nr* database.

Figure 1 shows the workflow that takes place within MetaFunc. Paired-end and/or single-end sequencing reads are used as input in fasta or fastq format. If trimming and mapping are not enabled, reads are used as input to Kaiju and subsequent microbiome analyses (Figure 1a). If trimming is enabled, reads are trimmed for adapters and undergo quality controls using fastp. If mapping is enabled, either the trimmed reads or raw input reads are first mapped to a designated host genome using STAR. Unmapped reads after host mapping are then used as input to Kaiju. STAR results are then used to obtain host gene information (Figure 1b).

### Microbiome analysis

MetaFunc parses through Kaiju results and gathers taxonomy IDs of species for taxonomic characterisation per sample and their corresponding protein accession numbers, which are subsequently annotated with GO terms (Figure 1a).

#### Taxonomy

Each classified read matches to a taxonomy ID in Kaiju. MetaFunc gathers the species level matches and adds up the raw reads matching to each species taxonomy ID. In cases of strain level identification, MetaFunc adds this count to its parent species. It also obtains scaled read counts in percentages by dividing the final read count of each taxonomy ID by the total reads that have mapped to species-level taxonomies (then multiplying by 100). For a dataset, the pipeline removes any taxonomy ID that is less than 0.001% in abundance in all samples of the dataset; this filter removes thousands of species that are likely to be false positives while retaining more confident classifications. Any remaining false classifications are thought not to affect downstream analyses, as the levels would be too low to impact true abundance (Ye et al., 2019), however, this value can be adjusted by the user. The taxonomy IDs that have passed the cutoff are then used in subsequent analyses. It should be noted that the pipeline still uses the original scaled percent abundances even after filtering. The pipeline would also include the lineage of the taxonomies using TaxonKit (Shen and Ren, 2021).

For a dataset, the MetaFunc pipeline outputs two tables containing species as rows and samples as columns with values being raw read counts or percent abundance for each species in the samples. If the user wishes to compare groups or conditions (eg. disease state vs. control), the pipeline calculates the average percent abundance of species among samples belonging to a group and this table is also given as an output. Differential abundance of microbes between groups is also carried out in MetaFunc using edgeR (McCarthy et al., 2012; Robinson et al., 2010). Raw read count tables are first filtered using the function *filterbyExpr* with threshold of 1, which is user-adjustable, and normalisation factors are calculated by *calcNormFactors* with default settings. *exactTest* is then applied to calculate differential abundance with *p*-values adjusted using Benjamini and Hochberg correction or false discovery rate (FDR).

#### Proteins

Kaiju outputs the accession number(s) of the protein match(es) with the highest BLOSUM62 alignment score of the read after translation into six open reading frames (ORF). It is possible to have more than one best protein match if two or more protein matches have equal scores in Kaiju. In order to account for this, we use proportional read counts per protein accession number where one read is divided by the number of best protein matches it has. Similar to that for taxonomy IDs, the pipeline adds up the proportional read counts per protein accession number of a species. Scaled reads as percent abundances are obtained by dividing the proportional count of each accession number by the total read counts that have mapped to a species (then multiplying by 100).

#### GO: database construction

MetaFunc relies on Kaiju’s nr_euk database for its taxonomic identification and corresponding protein matches. The nr_euk database is built on a subset from NCBI BLAST *nr* database containing Archaea, Bacteria, Fungi, Viruses, and other Microbial Eukaryotes (see https://raw.githubusercontent.com/bioinformatics-centre/kaiju/master/util/kaiju-taxonlistEuk.tsv). Identical sequences in the *nr* database are compiled into one entry and Kaiju only outputs the first protein accession number of an entry that has multiple identical sequences (Menzel et al., 2016). Thus, we needed to construct the protein-to-GO database such that all functional terms of any protein compiled in one *nr* entry are considered.

To facilitate GO annotations, we constructed an sqlite database in which GO annotations of a protein accession number from Kaiju can be looked up. We first gathered relevant NCBI *nr* database entries, converted all of the proteins of an *nr* entry into UniProt (Huang et al., 2011; The UniProt Consortium, 2017) entries, and then gathered corresponding GO annotations using the Gene Ontology Annotation (GOA) database for all those proteins (Camon et al., 2004). All GO annotations of one *nr* entry are then linked to the first protein of that entry in an sqlite database, which is used to annotate Kaiju protein accession matches with GO IDs. For more detailed information, please see the Notes section of the pipeline’s documentation page (https://metafunc.readthedocs.io/en/latest/notes.html). For MetaFunc, we provide pre-made databases for download (Sulit et al., 2021a, 2021b) but users can make their own updated databases following instructions from https://gitlab.com/schmeierlab/metafunc/metafunc-nrgo.git.

#### GO: protein annotation

For each sample, the pipeline obtains only the proteins that are from taxonomy IDs that passed cutoffs in the section “Taxonomy” described above. Their scaled proportional read counts, as in the section “Proteins” above, are still scaled against the total number of reads that mapped to a species. In order to compare groups or conditions, the pipeline first calculates the average of the corresponding proportional reads and scaled proportional reads of a protein accession number among samples of a group. It then searches for the GO terms annotating the (*nr*) protein using the created sqlite database described in *GO: Database Construction.* Each GO term set annotating an accession number is then updated by accessing parent terms related to the GO terms by “*is_a*” or “*part_of*” using *GOATOOLS* (Klopfenstein et al., 2018). Note that this update takes the entire set of GOs annotating the accession number into consideration such that no GO terms or path/s to the top of the GO directed acyclic graph (DAG) is doubled. GOATOOLS also parses other information regarding the go term such as description, namespace, and depth through the go-basic.obo file (Ashburner et al., 2000). The proportional and scaled read counts are then added to all GO terms annotating a protein, including updated terms. Finally, the percentage of reads covering a GO term within a namespace (Biological Process, Molecular Function, and Cellular Component) is calculated by dividing the scaled read count of a GO term by the total scaled read counts covering a namespace and multiplying by 100. The final output table of the pipeline is a contingency table with GO IDs of all namespaces as rows and samples or groups as columns, with percentage within a namespace as values.

#### Visualisation

To facilitate the exploration of results from MetaFunc, MetaFunc automatically builds an R shiny application, such that users can view and interact with the taxonomy and GO tables. The application allows users to select GO terms and identify the species whose proteins are annotated with the searched for term. Conversely, users may search for a species and obtain all GO terms associated with the searched for species. See the pipeline’s documentation page for more information (https://metafunc.readthedocs.io/en/latest/rshiny.html).

### Host analyses

Many microbiome communities are often associated with a host genome (Figure 1b). Reads belonging to the host genome have the capacity to misclassify as microbiome (Ye et al., 2019) and filtering of host reads has been a part of many microbiome studies, either prior to sequencing or *in silico* (Hugerth and Andersson, 2017; Macklaim and Gloor, 2018; Xia et al., 2018). The MetaFunc pipeline offers the option of mapping reads to a host genome using the programme STAR and using the unmapped reads from this step as input to Kaiju for the microbiome analysis.

MetaFunc also allows additional analyses of host reads after STAR mapping. Host genes are quantified using featureCounts (Liao et al., 2014) of the subread package. If comparisons between groups are indicated, edgeR is used to perform differential gene expression analysis (DGEA). Additionally, supplying a gene matrix transposed (*.gmt*) file from, for example, the molecular signatures database (GSEA, n.d.; Liberzon et al., 2011; Subramanian et al., 2005) allows for gene set enrichment analysis (GSEA) of host genes using the clusterProfiler package (Yu et al., 2012).

#### Host gene–microbe species correlation

When a comparison between groups is specified, the pipeline also performs Spearman correlation analysis between the top most significant differentially expressed genes (DEGs), expressed as transcript per million (TPM), and top most significant differentially abundant (DA) microbes, expressed as percent abundance. Results of these correlations are summarised in a matrix on which hierarchical clustering is performed and a heatmap is generated using Clustergrammer (Fernandez et al., 2017). Through this heatmap and table, a user can investigate the strength of correlation (*rho*) between a DA microbe and a DEG, and which microbes and genes have similar patterns of correlations.

### Tutorial/manual

For a more detailed description of the workflow, usage instructions, and results, documentation of the MetaFunc pipeline may be found at https://metafunc.readthedocs.io/en/latest/index.html.

## Illustration of tool use

### Dataset PRJNA413956: matched colorectal cancer and adjacent non-tumour tissue

In order to demonstrate the utility of the MetaFunc pipeline, we obtained publicly available transcriptomics data from the study of Li et al. (2018) consisting of 10 tumours and corresponding adjacent non-tumour colorectal tissue samples. Raw sequencing data were downloaded from https://www.ncbi.nlm.nih.gov/geo/query/acc.cgi?acc=GSE104836 and input to the pipeline and the full workflow carried out, generating data for host, microbiome, and host-microbiome correlation.

#### Microbiome results

##### Taxonomy

The MetaFunc pipeline outputs a table of percent abundances of species that are identified in each sample and an average of these abundances across members of the same group if a grouping condition is applied. We ran the pipeline with the intent of comparing microbiome species and function between colon cancer samples and non-tumour matched samples.

Previous studies have already established that certain microbes associate more with colorectal cancer (CRC) samples compared to healthy controls. We searched for *Fusobacterium nucleatum*, *Parvimonas micra,* and *Porphyromonas asaccharolytica* in the averaged group results. These microbes have previously been found to be more abundant in CRC cohorts in meta-analyses of several datasets (Dai et al., 2018; Thomas et al., 2019). We also searched for *Bifidobacterium* species, *Bifidobacterium bifidum* and *Bifidobacterium longum*; Bifidobacteria are thought to confer protection from CRC (Wei et al., 2018).

**Figure 2:**
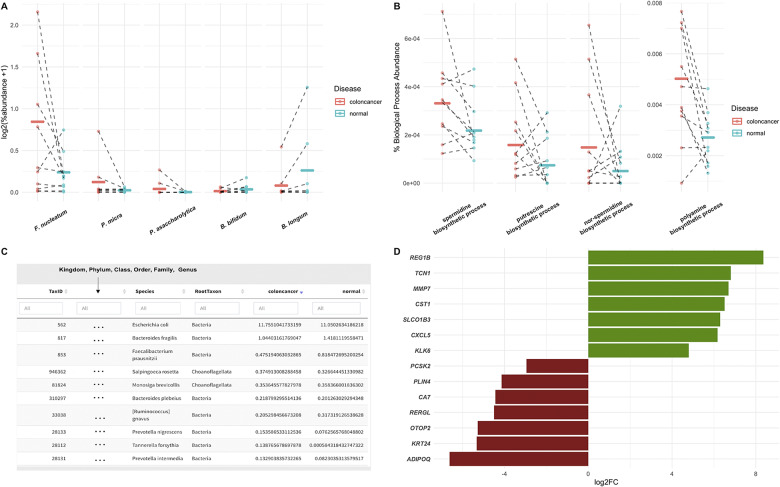
**MetaFunc Microbiome and Host Analyses of Dataset PRJNA413956. (a)** **Average percent abundance of selected bacterial species in CRC tissue compared to matched** **non** **-tumour** **(normal) samples.** From MetaFunc tabulated results, we plotted the percent abundances of selected bacteria in CRC and matched normal samples. Raw values were first log_2_ transformed, with prior addition of 1 as a pseudocount to account for 0 values. Individual points represent per sample transformed values in red (CRC) and blue (Normal). Per group means are represented by the horizontal lines. Dotted lines connect matched CRC and normal samples. **(b)** **Percent abundance of specific polyamine biosynthetic process GO terms among all biological process GOs in a sample/group compared between CRC (red) and normal (blue) samples.** Values were calculated as described in section *“*GO: protein annotation” and output in MetaFunc tables or in the R Shiny application. These values were plotted, overlaying group means (horizontal lines) and individual values (data points). **(c)** **Screenshot from MetaFunc R shiny application.**This view shows the first 10 species with proteins contributing to the GO *polyamine biosynthetic process.* The R Shiny application columns include a URL (not shown in screenshot), which is linked to the NCBI’s Taxonomy Browser, the Species Taxonomy ID, Lineage (indicated as “*…*” in screenshot), Root Taxon, and percent abundances of the species in the two groups being compared: CRC and normal samples. Note that percent abundances refer to the total abundance of the species in question, not just the proteins contributing to the GO term. Results shown are sorted from highest to lowest percent abundance in the colon cancer cohort. **(d)** **Fold change of representative upregulated and downregulated human genes** (Li et al., 2018) between CRC and matched normal samples in this study. Fold change values were obtained from the edgeR results of the pipeline. All these genes are significant (FDR < 0.05) in both this study and the source publication.

The bars in Figure 2a show the average percent abundance of the species between samples from tumour and matched non-tumour tissue as identified through MetaFunc. As MetaFunc provides a per sample data, we are also able to plot individual values of CRC (red) and matched normal (blue) samples.

As seen in Figure 2a, MetaFunc identified *F. nucleatum, P. micra,* and *P. asaccharolytica* as being relatively more abundant (ie. have higher average percent abundance) in the CRC group while the *Bifidobacterium* species are relatively more abundant in the normal group.

MetaFunc also has a step that utilises edgeR to perform differential abundance on per sample species read counts, stratified according to CRC and non-tumour grouping. This resulted in a total of 117 species that were significantly different between the groups (FDR < 0.05). There are 59 species upregulated and 58 downregulated in colon cancer samples. Through the MetaFunc results, we identified *Tanerella forsythia* as the most prominent enriched species in the colon cancer cohort with a log_2_ FC = 7.40. *T. forsythia* is a known oral pathogen, thought to be part of the so-called Red complex of periodontal pathogens, along with *Porphyromonas gingivalis*, and *Treponema denticola* (Malinowski et al., 2019). Members of this Red Complex have been found to be enriched in subtype CMS1 of CRCs (Purcell et al., 2017), the subtype most associated with immune process activation in CRC (Dienstmann et al., 2017; Guinney et al., 2015; Inamura, 2018).

##### Function

MetaFunc is intended to enable comparisons of the functional potential of the microbiome between groups. MetaFunc uses GO annotations of protein matches from Kaiju. To demonstrate, we focused on polyamine biosynthetic processes GO terms. Polyamines (PAs) are polycations found to play important biological functions in cell growth. These molecules have been found to be associated with tumour progression and growth (Gerner and Meyskens, 2004; Soda, 2011; Tofalo et al., 2019). Although cells are able to biosynthesize polyamines and even export them, a large source of cellular polyamines comes from uptake from their surroundings and, importantly, the microbiota is thought to be an essential source (Soda, 2011; Thomas et al., 2019; Tofalo et al., 2019) with spermidine and putrescine being the most common of bacterial PAs (Tofalo et al., 2019).

The bars in Figure 2b show the percent of reads among biological process GOs covering PA biosynthetic processes in the CRC and Normal conditions, superimposed with the individual values of samples from the CRC (red) and Normal (blue) groups. From Figure 2b, we saw that several of the polyamine biosynthetic processes were relatively more abundant (ie. higher percent of reads among biological process GOs) in the CRC cohort compared to the normal cohort, using protein annotations.

We used the built-in MetaFunc shiny application to facilitate an inquiry into the microbes species that may contribute to polyamine synthesis. To illustrate, we searched for “*polyamine biosynthetic process*” in the “GO to TaxIDs” tab of the application, and obtained a total of 126 TaxIDs contributing to the GO term in both CRC and normal samples. Of these TaxIDs, we identified *Escherichia coli* and *B. fragilis* to be most abundant in both cohorts. However, differences in the relative abundance of some microbial species can be identified between cancer and normal cohorts, notably several of which are oral pathogens from the genus *Prevotella.* A striking difference in abundance was seen in *T. forsythia,* which was previously found to be significantly more abundant in the CRC cohort via edgeR (Figure 2c). These data suggest that *T. forsythia* represents one of the bacterial species that most contributes to increased polyamine synthesis in CRC samples in this cohort.

#### Host results

The dataset we used for this study was from a total RNA transcriptomics run aiming to identify long non-coding RNAs (lncRNAs) and mRNAs in CRC samples (Li et al., 2018). Therefore, we first mapped the reads to the human genome using the STAR mapping utility of the pipeline, subsequently using only the unmapped reads for the microbiome analyses. From the reads mapped to the human genome, MetaFunc was able to obtain counts of reads covering human genes and using these, obtained DEGs between CRC and matched normal samples through edgeR. MetaFunc results showed a total of 1,476 DEGs with an FDR < 0.05 and |log_2_fold change| > 2. From these, we found all the top 5 upregulated and top 5 downregulated genes as reported in the source publication (Li et al., 2018), as well as all the genes they had randomly selected for expression confirmation via qPCR. Figure 2d shows their fold change as found through MetaFunc.

MetaFunc is also able to perform host gene set enrichment analysis using the DEGs. Significant gene sets (*p.*adjust < 0.05) with the highest normalised positive enrichment scores (NES) included such terms as ribosome biogenesis, DNA replication, mitotic nuclear division, and condensed chromosome (see Supplementary Table S1), many of which appear to be related to cell division or replication, consistent with the findings of the source publication (Li et al., 2018), that the upregulated lncRNAs they found were involved in mitosis, cell cycle process, and mitotic cell cycle.

#### Host–microbiome correlations

We set MetaFunc’s default abundance cutoff for microbial identification to 0.001% to remove most probable contaminants and so as not to lose any other meaningful taxonomies. It has been shown in a prior study (Ye et al., 2019), however, that most classifiers call false positives at below 0.01% abundance. We, therefore, applied this 0.01% cutoff in looking at the host–microbiome correlations in this dataset to narrow our focus on microbes that are more likely to be involved in our test case.

In using the 0.01% cutoff, MetaFunc was able to only identify 19 DA microbes. Their correlations with the top 100 significantly abundant genes can be seen at the URL: http://amp.pharm.mssm.edu/clustergrammer/viz/5f02a49e8ec9bb33170b865c/cor.deg-tax.matrix.tsv. Table 1 highlights some notable correlations between DA microbes and differentially expressed human genes. *T. forsythia*, although significantly abundant in CRC samples, do not correlate significantly with any DEGs in CRC. Among its highest correlations, however, included the gene Colorectal Neoplasia Differentially Expressed (*CRNDE*).

**Table 1.** Spearman correlation between DA microbes and DGEs in CRC.

*Gene name*	Gene ID	TaxID	Species	rho	*p*-value
*CRNDE*	ENSG00000245694.10	28112	*Tannerella forsythia*	0.29	0.22
		36911	*Clavispora lusitaniae*	0.70	0.00063
		106590	*Cupriavidus necator*	0.65	0.0019
		1314	*Streptococcus pyogenes*	0.63	0.0027
*TCN1*	ENSG00000134827.8	106590	*Cupriavidus necator*	0.71	0.00042
		36911	*Clavispora lusitaniae*	0.61	0.0045
		1314	*Streptococcus pyogenes*	0.60	0.0048
*WNT2*	ENSG00000105989.10	1314	*Streptococcus pyogenes*	0.84	4.07E-06
		106590	*Cupriavidus necator*	0.75	0.00015
		36911	*Clavispora lusitaniae*	0.75	0.00016

Conversely, we investigated which species correlated with *CRNDE*. The highest correlations were with microbes *Candida lusitaniae, Cupriavidus necator,* and *Streptococcus pyogenes.* All correlations were determined to be significant. The same species were among the highest correlations of *TCN1*, and *WNT2*. *TCN1* was among the top DEGs in cancer identified in this study as well as in the source publication (Li et al., 2018). *WNT2* meanwhile is part of the Wnt/β-catenin pathway, which has roles in cell proliferation, cell migration, and cell differentiation. *WNT2* is responsible for the hyperactivation of β-catenin and is known to be upregulated in CRC (Jung et al., 2015).

### Dataset PRJNA404030: consensus molecular subtypes of CRC samples

To illustrate MetaFunc’s capacity to compare more than two sample groups, we used MetaFunc to analyse transcriptome reads from the study of Purcell and colleagues (Purcell et al., 2017) (raw reads may be accessed at https://www.ncbi.nlm.nih.gov/bioproject/PRJNA404030), which are grouped into four CRC consensus molecular subtypes (CMS). A total of 33 samples were collected during surgical resection of tumours, and sample preparation for RNA sequencing was carried out using the Illumina TruSeq Stranded Total RNA Library preparation kit. For these samples, fastq-mcf from ea-utils (Aronesty, 2011, 2013) and SolexaQA++ (Cox et al., 2010) were used to trim reads, which were then run through Salmon (Patro et al., 2017) to quantify transcript expression. The publicly available CRC CMS classifier (Guinney et al., 2015) was used to categorise samples into one of four CMSs. Of the 33 samples, only 27 were classified into a CMS and of these, only one sample was classified into CMS4. This sample was also removed from the dataset for lack of replicates leaving a total of 26 samples – 7 samples in CMS1, 11 in CMS2, and 8 in CMS3. Metafunc was used with default parameters, except for the following options: trimming was set to false, and featureCounts with reverse stranded option was used.

#### Microbiome results

##### Taxonomy

**Figure 3:**
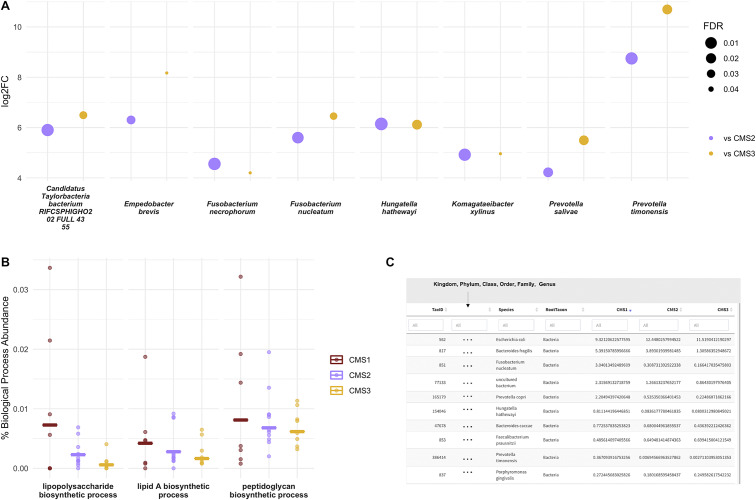
**MetaFunc Microbiome Analysis of Dataset PRNJA4040030. (a)** **Microbes that are significantly more abundant (FDR < 0.05) in CMS1 compared to CMS2 (purple) and CMS3 (yellow).** Microbes are considered DA in CMS1 if it is identified through edgeR as DA in both CMS1 versus CMS2 and CMS1 versus CMS3 comparisons. Log_2_FC (*y*-axis) is the log_2_ of the fold-change between CMS1 and the other subtypes (eg. CMS1/CMS2); FDR (point sizes) is the false discovery rate adjusted *p*-values. **(b)** **Percent abundance of specific PAMPs biosynthetic process GO terms among all biological process GOs in a sample/group compared between CRC subtypes, CMS1 (red), CMS2 (purple), and CMS3 (yellow).** Values were calculated as described in section “GO: protein annotation” and output in MetaFunc tables or in the R Shiny application. These values were plotted, overlaying group means (horizontal lines) and individual values (data points). **(c)** **Screenshot of R shiny application showing the relative abundances of species associated with PAMPs biosynthetic processes compared among CMS1, CMS2, and CMS3.** This view shows the first 10 species, with the highest abundances in CMS1, with proteins contributing to any of the PAMPs biosynthetic processes described above. The application columns show a URL (not shown in screenshot), which is linked to the NCBI’s Taxonomy Browser, the Species Taxonomy ID, Lineage (shown as “…” in screenshot), Root Taxon, and percent abundances of the species in the three groups being compared: CMS1, CMS2, and CMS3. Note that percent abundances refer to the total abundance of the species in question, not just the proteins contributing to the GO term. Results shown are sorted from highest to lowest percent abundance in the CMS1 group.

MetaFunc performed pairwise differential abundance analysis on the three groups using edgeR. From MetaFunc’s results, we considered a species to be significantly abundant in a subtype if it is significantly abundant compared to both of the other subtypes. For instance, a significantly abundant species in CMS1 must be significantly abundant in the CMS1 versus CMS2 and CMS1 versus CMS3 comparisons. Using this definition, only CMS1 had species that were significantly abundant (FDR < 0.05) compared to both CMS2 and CMS3. Figure 3a shows the false discovery rate (FDR; diamonds) and log_2_ fold change (bars) of the species in CMS1 compared to CMS2 (blue) and CMS3 (brown).

We take note of species in the genera *Prevotella* and *Fusobacterium,* which have previously been associated with CRC. *Fusobacterium nucleatum* in particular has strong evidence of an association with CRC (Dai et al., 2018; Gao et al., 2015; Ye et al., 2017). Most of these are also members of the oral microbiota, which have also previously been associated with cancer development particularly through inflammatory processes (Whitmore and Lamont, 2014). We found no species that were significantly abundant in CMS2 or CMS3 using the given criteria.

##### Function

Through the microbiome functional results of MetaFunc, we then investigated if processes relating to pathogen-associated molecular patterns (PAMPs) were contributed by the microbial communities, considering that CMS1 is characterised by immune responses, which are usually triggered when the human immune system recognises such molecules. We used the MetaFunc R shiny application to search for terms “*lipopolysaccharide biosynthetic process*,” “*lipid A biosynthetic process*” and “*peptidoglycan biosynthetic process*,” and their relative abundances. Unsurprisingly, all PAMPs were relatively more abundant in CMS1 (Figure 3b).

Using the MetaFunc R shiny application, we also searched for which species might be contributing to the above terms. Figure 3c is a screenshot of the application showing the species contributing to any of the terms in Figure 3b. Figure 3c is arranged from highest to lowest relative abundance in CMS1 and we saw microbes that were among those identified to be significantly abundant in CMS1 such as *F. nucleatum, Hungatella hathewayi, and Prevotella* species. These microbes have previously been associated with CRC (Dai et al., 2018; Gao et al., 2015; Wirbel et al., 2019; Ye et al., 2017).

#### Host results

##### Gene set expression analysis

MetaFunc calculated DEGs between subtypes in a pairwise manner (ie. CMS1 versus CMS2, CMS1 versus CMS3, CMS2 versus CMS3). From the DEGs of the results, MetaFunc was also able to calculate enriched gene sets for each comparison. Similar to identifying DA microbes, we obtained a final set of enriched gene sets for a subtype if it showed enrichment compared to both other subtypes (*p*.adjust < 0.05). Unsurprisingly, we saw several host GO terms involved in immune response enriched in CMS1, including regulation of innate immune response, response to interferon gamma, and positive regulation of cytokine production among others. Enriched host GOs in CMS2 are involved in the cell cycle and ribosome biogenesis, with terms such as tRNA metabolic process, ribosomal large subunit biogenesis, and DNA replication initiation, while host GOs enriched in CMS3 involve metabolic processes, for example, primary xenobiotic metabolic process, flavonoid metabolic process, and lipid catabolic process. These results are consistent with the description of these three CRC subtypes in the original CMS study (Guinney et al., 2015). The top enriched gene sets for each subtype can be found in Supplementary Tables S2–S7.

#### Host–microbiome results

Next, using correlation results from MetaFunc, we investigated which of the top significantly DEGs correlated with the significantly abundant microbes in CMS1. We obtained the following statistically significant correlations between host and microbiome abundances shown in Table 2.

**Table 2.** Spearman correlation between DA microbes in CMS1 and DGEs in CMS1.

*Gene name*	Gene ID	TaxID	Species	rho	*p*-value
*WARS1*	ENSG00000140105.18	1802307	*Candidatus Taylorbacteria bacterium RIFCSPHIGHO2_02_FULL_43_55*	0.59	0.0015
*WARS1*	ENSG00000140105.18	851	*Fusobacterium nucleatum*	0.55	0.0035
*RNF213*	ENSG00000173821.19	1802307	*Candidatus Taylorbacteria bacterium RIFCSPHIGHO2_02_FULL_43_55*	0.54	0.0048
*ICAM1*	ENSG00000090339.9	1802307	*Candidatus Taylorbacteria bacterium RIFCSPHIGHO2_02_FULL_43_55*	0.50	0.01
*RNF213*	ENSG00000173821.19	851	*Fusobacterium nucleatum*	0.50	0.01
*PARP14*	ENSG00000173193.15	851	*Fusobacterium nucleatum*	0.47	0.02
*PARP14*	ENSG00000173193.15	1802307	*Candidatus Taylorbacteria bacterium RIFCSPHIGHO2_02_FULL_43_55*	0.47	0.02
*PARP9*	ENSG00000138496.16	851	*Fusobacterium nucleatum*	0.46	0.02
*ICAM1*	ENSG00000090339.9	851	*Fusobacterium nucleatum*	0.46	0.02
*SLC15A3*	ENSG00000110446.11	1802307	*Candidatus Taylorbacteria bacterium RIFCSPHIGHO2_02_FULL_43_55*	0.46	0.02
*STAT1*	ENSG00000115415.19	386414	*Prevotella timonensis*	0.44	0.02
*CD163*	ENSG00000177575.12	1802307	*Candidatus Taylorbacteria bacterium RIFCSPHIGHO2_02_FULL_43_55*	0.42	0.03
*PARP14*	ENSG00000173193.15	386414	*Prevotella timonensis*	0.42	0.03
*CD163*	ENSG00000177575.12	386414	*Prevotella timonensis*	0.42	0.03
*ICAM1*	ENSG00000090339.9	386414	*Prevotella timonensis*	0.41	0.04
*PARP9*	ENSG00000138496.16	1802307	*Candidatus Taylorbacteria bacterium RIFCSPHIGHO2_02_FULL_43_55*	0.41	0.04
*CD163*	ENSG00000177575.12	851	*Fusobacterium nucleatum*	0.41	0.04
*SLC15A3*	ENSG00000110446.11	851	*Fusobacterium nucleatum*	0.40	0.04
*STAT1*	ENSG00000115415.19	851	*Fusobacterium nucleatum*	0.40	0.04
*PML*	ENSG00000140464.20	1802307	*Candidatus Taylorbacteria bacterium RIFCSPHIGHO2_02_FULL_43_55*	0.39	0.05
*GBP1*	ENSG00000117228.10	28448	*Komagataeibacter xylinus*	−0.39	0.05
*CEBPA*	ENSG00000245848.3	154046	*Hungatella hathewayi*	−0.41	0.04
*GNLY*	ENSG00000115523.16	28448	*Komagataeibacter xylinus*	−0.43	0.03

Some of these correlations may be found in http://maayanlab.cloud/clustergrammer/viz/610d8b3c97f268000ea37f41/cor.deg-tax.matrix.tsv. This is the hierarchical cluster obtained when correlating top DA microbes and top DGEs in CMS1 compared to CMS2. It is to be noted that there may be correlations in this clustering that are not found in CMS1 compared to CMS3 and are therefore not reported in Table 2.

The Spearman correlations (rho) between DA microbes and DEGs were quite small in value (the highest value being ~ |0.59| between *WARS1* and *Candidatus Taylorbacteria bacterium RIFCSPHIGHO2_02_FULL_43_55*). Nevertheless, several of the genes appeared to have a relevant function with regards to CRC and immune responses. Table 3 shows information for genes that correlated with *Fusobacteria* and *Prevotella* species in our analyses. These two microorganisms have previously been associated with CRC.

**Table 3.** Gene Information of DEGs correlated with DA Microbes in CMS1.

*Gene name*	Protein name	Relevant protein/gene function	Association with CRC and/or inflammation	Sources
*ICAM1*	Intercellular adhesion molecule 1	Mediates cell adhesion of cytotoxic T lymphocytes and natural killer cells	Upregulation of ICAM1 inhibits tumour growth and metastasis; a soluble form (sICAM1) is increased in CRC tissues compared to normal, and is associated with an inflammatory tumour microenvironment	Sánchez-Rovira et al., 1998; Schellerer et al., 2019; Tachimori et al., 2005
*SLC15A3*	Solute carrier (SLC) 15A3	Membrane transporter; highly expressed in macrophage populations	Upregulated by LPS via NF-κβ pathway; influences pro-inflammatory cytokine production triggered by TLR-4	Song et al., 2018; Wang et al., 2014
*CD163*	CD163 receptor	M2 Macrophage marker	M2 macrophages are anti-inflammatory macrophages and CD163+ tumour-associated macrophages are with mesenchymal transition and poor prognosis in CRC; are correlated with CCL4	Argyle and Kitamura, 2018; Bayoumi et al., 2016; De la Fuente López et al., 2018; Pinto et al., 2019
*STAT1*	Signal transducer and activator of transcription 1	Transcription factor for IFN signalling	Upregulated in CRCs; correlated with PD-L1 and PD1 immune checkpoint inhibitors; pro-oncogenic in MSI CRCs	Leon-Cabrera et al., 2018; Tanaka et al., 2020
*PARP 9*	Poly(ADP-ribose) polymerase family member 9	Involved in cell migration	Possible role in metastasis	Vyas and Chang, 2014
*PARP 14*	Poly(ADP-ribose) polymerase family member 14	Involved in IL-4 signalling and cell migration	Involved in anti-apoptotic effects	Vyas and Chang, 2014
*RNF213*	Ring finger protein 213	Involved in PI3K-AKT pathway for cell growth	Involved in endothelial angiogenesis	Ohkubo et al., 2015
*WARS1*	Tryptophanyl-TRNA synthetase 1	Inhibitor of angiogenesis; Involved in IFN-g signalling	Involved in immune responses; cleaved form potentially inhibits angiogenesis; increased levels indicate better CRC survival	Ghanipour et al., 2009; Jin, 2019

### Comparison of MetaFunc results to HUMAnN2

HUMAnN2 (Franzosa et al., 2018) is one of the packages most frequently used to assess functional pathways of the microbiome, and to determine which organisms are contributing to the functional pathways. HUMAnN2 works by pre-screening which taxonomies are present in a sample using MetaPhlAn2, afterwards aligning the reads to pangenomes of the classified taxonomies for gene hits. Unclassified reads then undergo an organism-agnostic translated search (Franzosa et al., 2018). MetaPhlAn2 has a rather limited database for the pre-screening of organisms (Ye et al., 2019), resulting in a high level of unmapped reads and a limited number of organisms identified.

We ran the same sequencing reads from the study PRJNA413956 (Li et al., 2018) through HUMAnN2, first trimming with fastp and removing human-mapped reads using the same conditions as for the MetaFunc pipeline. To be more comparable, we changed the pre-screen threshold of HUMAnN2 to 0.001% of mapped reads. Part of HUMAnN2’s tiered search uses diamond (Franzosa et al., 2018), which requires higher memory and run time compared to Kaiju, used by MetaFunc (Ye et al., 2019). From taxonomy identification, using Kaiju, to the generation of GO tables, took MetaFunc 11.39 hours to complete, while a comparable analysis using HUMAnN2 took 65.9 hours to complete, almost six times slower than MetaFunc on the same machine (CentOS Linux release 7.9.2009). Notably, HUMAnN2 has an additional pathway abundance and pathway coverage analysis absent from MetaFunc. Runs for HUMAnN2 may be accessed at https://github.com/asulit08/Humann2_PRJNA413956.

Results showed that for the 20 samples analysed, 8.4–22.9% of reads were mapped after nucleotide and protein alignment steps. In contrast, using Kaiju in the MetaFunc pipeline resulted in 33.8–56.2% reads mapped to microbial species through protein matches. We also detected only 87 species across the 20 samples using HUMAnN2, compared with a total of 4,267 species using Kaiju in the MetaFunc pipeline. Further, HUMAnN2 was only able to detect Bacteria and Viruses in the samples, while MetaFunc analysis was able to detect Fungi and Archaea as well. We also investigated the concordance of the microbial GO terms that had been classified to a taxonomy from the MetaFunc run with that of HUMAnN2. We focused on only the Bacteria and Viruses – related GO terms as found in the HUMAnN2 run. We found that the majority (69–100%) of the GOs found in HUMAnN2 was also found in the MetaFunc run. There were more unique GO terms found in the MetaFunc run, which may be due to the higher number of species detected with MetaFunc (Supplementary Figure S1).

We investigated the same species and polyamine (PA) biosynthetic process GO terms in our HUMAnN2 results as we had in the MetaFunc run of dataset PRJNA413956 (Supplementary Figures S2 and S3). We see in Supplementary Figure S2 that abundances of the species in CRC and normal groups have the same trends in HUMAnN2 results as in that of MetaFunc (Kaiju) results. In HUMAnN2 runs, however, we were not able to find *B. bifidum* among the identified species in the PRJNA413956 cohort. Meanwhile, we also see the same trends in the abundances of PA biosynthetic process GO terms in CRC samples compared to matched normal samples in HUMAnN2 runs, as in our MetaFunc run (Supplementary Figure S3), except for nor-spermidine biosynthetic process, which was not seen using HUMAnN2. Differences in abundance values were noted when comparing individual samples, however, direct comparison between HUMAnN2 and MetaFunc is difficult as raw read-counts scaled to species-classified reads are used in MetaFunc, while HUMAnN2 uses reads-per-kilobase (RPK)-based relative abundances.

## Discussion

MetaFunc allowed us to investigate the relative abundances of known CRC – associated bacteria between CRC samples and matched normal tissues using the PRJNA413956 dataset. MetaFunc results show that the average abundance of microbes known to contribute to CRC progression are higher in cancer samples while those protective against CRC have higher average abundance in normal samples. Through MetaFunc, we also identified that *Tannerella forsythia*, a known oral pathogen and part of the Red Complex that causes periodontal diseases (Malinowski et al., 2019), is significantly more abundant in CRC tissues than in normal tissues. Oral pathogens have previously been seen to associate with CRC samples (Flemer et al., 2018; Koliarakis et al., 2019; Thomas et al., 2019; Whitmore and Lamont, 2014). By investigating the R shiny application from MetaFunc, we also found that *T. forsythia,* along with bacteria in the *Prevotella* genera, contributed to polyamine biosynthetic processes indicating that some oral pathogens contribute to cancer progression by producing polyamines that could be taken up by the surrounding cells.

Furthermore, we were able to find known bacteria in the MSI-Immune subset of CRCs by identifying the DA microbes in CMS1 compared to both CMS2 and CMS3 subtypes, as identified by MetaFunc’s edgeR step. *Fusobacteria* have long been associated with CRC development (Dai et al., 2018; Gao et al., 2015; Thomas et al., 2019; Ye et al., 2017) while *Prevotella* includes species that inhabit the oral cavity; there have also been *Prevotella* species that were found to be abundant in CRC cohorts (Dai et al., 2018; Flemer et al., 2018; Gao et al., 2015). In line with this, PAMPs were also found to be relatively more abundant in the CMS1 cohort upon investigation through MetaFunc’s R shiny application. The involvement of these bacteria in CMS1 as well as a relatively higher abundance of proteins contributing to biosynthesis of PAMPs in CMS1 indicate a role of microorganisms in the immune responses that drive the development of CRC in these tumours. This is further supported by correlation with host genes involved in inflammation and/or CRC development as found using MetaFunc’s spearman correlation step. The lack of significantly abundant microorganisms in CMS2 and CMS3 may reflect that the CRC development in these subtypes are not as dependent on immune dysregulation.

We created MetaFunc with the aim of identifying microbes and their functional contribution in a microbiome environment. One of the most widely used packages for this is HUMAnN2 (Franzosa et al., 2018) but we find the taxonomic identification generated by HUMAnN2 to be limited, because of its reliance on marker genes. For our purposes, we found MetaFunc invaluable for investigating novel microbes that did not have marker gene representation, in addition to being faster for larger amounts of data, and compatible with downstream analysis programs. We showed in this paper that results from the pipeline are biologically meaningful and corroborate previous literature. It was meant to be an alternative or a complement to HUMAnN2 in this regard. Although similar trends were seen in taxa and gene ontologies of interest between CRC and matched normal samples, fewer test reads were designated as taxa using HUMAnN2 compared to MetaFunc in our comparative analysis. Unfortunately, direct comparison was not possible because HUMAnN2 and MetaFunc use different abundance outputs.

We acknowledge that, especially at the 0.001% abundance cutoff, some of these species we are seeing could be false positives, or that these could be contaminants from sequencing and processing kits used (Goffau et al., 2018; Salter et al., 2014). We would caution users in interpreting data from microbes of very low abundances and would recommend following the advice of including negative control samples in sequencing (Salter et al., 2014). Indeed we could be seeing these effects upon looking at the microbes correlating with significantly abundant host genes in CRC samples from PRJNA413956. While *C. lusitaniae* is an opportunistic pathogen causing candidemia (Desnos-Ollivier et al., 2011; Krcmery et al., 1999) possibly exploiting the lowered immune responses in cancer patients (Aslani et al., 2018), and some *Streptococcus* species have previously been implicated in CRC (Kumar et al., 2017; Xia et al., 2020), with *S. pyogenes* having been known to cause invasive infections in humans (Parks et al., 2015), *C. necator* (formerly known as *Ralstonia eutropha* (Reinecke and Steinbüchel, 2009), is a soil bacterium that may be a sequencing contaminant in this dataset. *Cupriavidus* and *Ralstonia* species have been previously identified as common contaminants in meta-omics studies (Guo et al., 2019; Salter et al., 2014).

MetaFunc analyses host and microbiome reads, providing a user-friendly, interactive R-shiny application to investigate results, most useful for those with candidate microbes and function in mind, or for exploratory analyses of the characteristics of a user’s dataset. It should be noted that these values are based on raw counts and percent abundances. Microbiome datasets are considered compositional (Gloor et al., 2017; Gloor and Reid, 2016), and this should be taken into consideration during further analysis. We reiterate that values shown in the shiny application (eg. average of microbial relative abundances within a group), are to be used as initial comparisons and description of the data, and care should be taken in its interpretation, especially in the light of compositional data analysis. Further downstream analysis, such as differential abundance of microbes, can also facilitate parsing of tables in the shiny application. A gold standard for differential abundance analysis in microbiome datasets is currently non-existent and different tools reach different results (Calgaro et al., 2020; Nearing et al., 2022). We offer edgeR in MetaFunc as we believe it is a good initial tool to explore DA microbes, though this is offset by being prone to false positives (Thorsen et al., 2016). MetaFunc results provide potential starting points for more in-depth analyses or hypothesis generation for experimental procedures. In this regard, we provide results in “*.tsv”* formats for use in other downstream bioinformatics applications, so users might apply their own analyses of choosing.

Correlation analysis on compositional data has the same contentious issue as differential abundance. Although there is published literature supporting the use of Spearman rank correlation coefficient in this analysis (Cremonesi et al., 2018; Dai et al., 2018; Geng et al., 2014), there are dissenting voices stating that there are spurious correlations, especially in compositional data (Aitchison, 1982; Faust et al., 2012; Friedman and Alm, 2012; Lovell et al., 2015; Pearson, 1897), and as such, conclusions from such correlations are meaningless (Lovell et al., 2015). Nevertheless, Spearman correlation serves a useful purpose, especially for an initial exploration of the data. Should users choose other analyses methods, intermediate results are provided with the pipeline.

This method was developed specifically for an RNA-seq (transcriptomic/metatranscriptomic) dataset, allowing for the common analysis applied to such studies. It is intended for an initial complete analysis of the data, with only a single configuration file and sample sheet necessary once installation of the tool has been done. Users can augment this analysis by accessing host gene and microbiome count files supplied by the pipeline and use this as input in other applications. Users can also potentially use the microbiome aspect of the pipeline on a metagenomic dataset, and can adjust this in the configuration file. As Kaiju (Menzel et al., 2016) identifies a single best protein match (or multiple matches with equal scores) of a read, we recommend its usage for short-read datasets. An exception could be made for long read sets in which the user is certain an input read will only span one protein.

We used the MetaFunc pipeline to compare genes and microbes between or among groups, but exploratory analyses of datasets from single groups can also be carried out.

While the methodology of this paper focuses on RNA sequences, metagenomic content could affect variation seen in microbial community gene expression (Franzosa et al., 2014). It should be noted that gene copy number, for instance, could affect transcript counts. Counts seen with metatranscriptomic data would also reflect a species’ gene expression contribution as opposed to abundance. It would be prudent to take this into consideration when interpreting biological implications of the results.

## Conclusion

Here we presented MetaFunc, a single pipeline for analysing host and microbiome sequencing reads and their relationships. We found that we identified more microbes in our test datasets using MetaFunc compared to HUMAnN2, while microbes and functions of interest were comparable between the two. We have used MetaFunc to determine that microbes previously known to have associations with CRC are indeed relatively more abundant in CRC samples compared to normal samples. Furthermore, we were able to use MetaFunc to highlight that these microorganisms could contribute to CRC progression through polyamine production.

For a dataset with more than two groups, we have also used MetaFunc to identify abundant bacteria in a CRC subtype associated with immune responses, while conversely, we have not been able to identify significant microbes in the other CRC subtypes. MetaFunc’s Spearman correlation step showed that the significant bacteria correlate with human DEGs that function in immune responses and CRC progression. We showed that MetaFunc was able to identify candidate microorganisms that differentiate sample groups and provide insight on the functional capacities of these candidates.

## Data Availability

MetaFunc is freely available through https://gitlab.com/schmeierlab/workflows/metafunc.git, and full documentation can be found in https://metafunc.readthedocs.io/en/latest/.

## Supplementary material

For supplementary material accompanying this paper visit https://doi.org/10.1017/gmb.2022.12. click here to view supplementary material
