# Supplementary material for: MetaFunc: taxonomic and functional analyses of high throughput sequencing for microbiomes
Source: Gut Microbiome (Camb). 2023 Jan 12;4:e4. doi: 10.1017/gmb.2022.12 (PMC11406379; doi:10.1017/gmb.2022.12)
Supplement: Supplementary file 1 [file S2632289722000123sup001.docx]

**Supplementary Material**

## **Supplementary Figures**

| 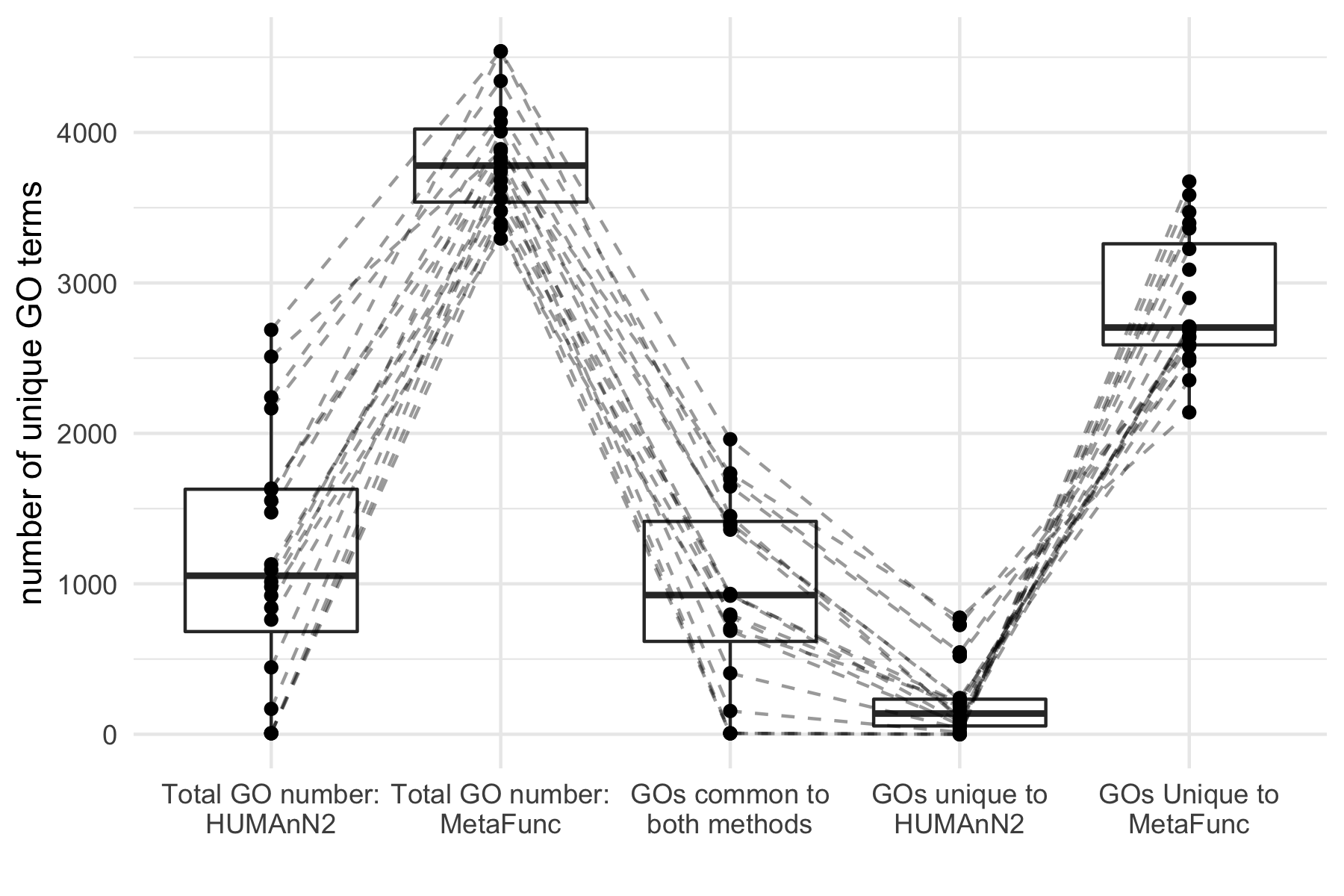 |
| --- |
| **Supplementary Figure S1. Comparison of the number of unique microbial GO terms between MetaFunc and HUMAnN2 from the analysis of the PRJNA413956 cohort.** We compared the number of GO terms from Bacteria and Viruses from MetaFunc with the number of microbial GO terms from HUMAnN2 that have been classified to a taxonomy. Dashed lines connect one sample from the PRJNA413956 cohort. |

| 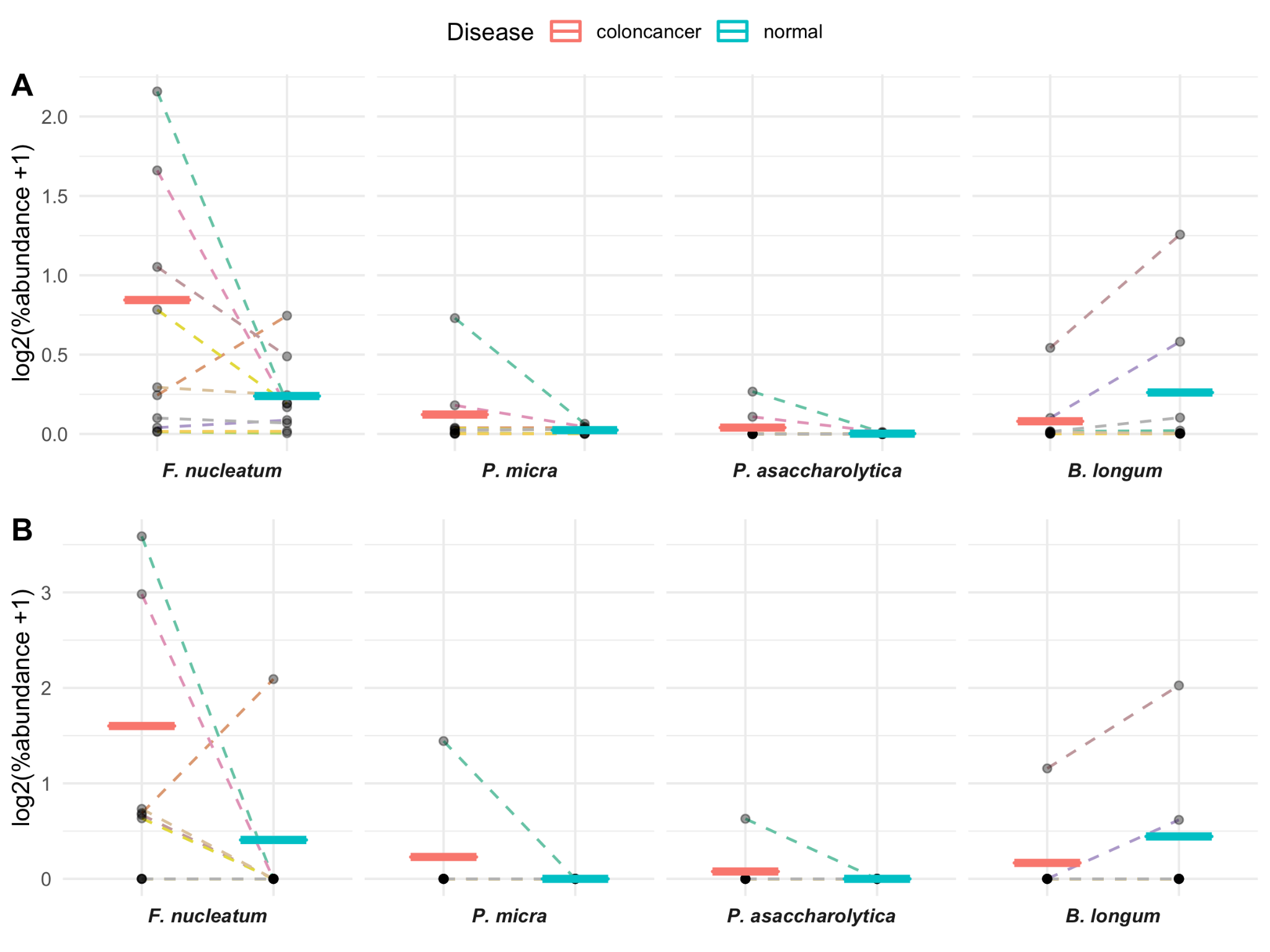 |
| --- |
| **Supplementary Figure S2. Average percent abundance of selected bacterial species in CRC tissue compared to matched non-tumor (normal) samples as measured by A.** MetaFunc (top) **or B.** HUMAnN2 (bottom)**.** We plotted the percent abundances of selected bacteria in CRC and matched normal samples. Raw values were first log_2_ transformed, with prior addition of 1 as a pseudocount to account for 0 values. Individual points represent individually transformed sample values. Per group means are represented as horizontal lines and colored according to disease state. Dashed lines connect matched CRC and normal values. Line colors correspond to individual patients. |

| 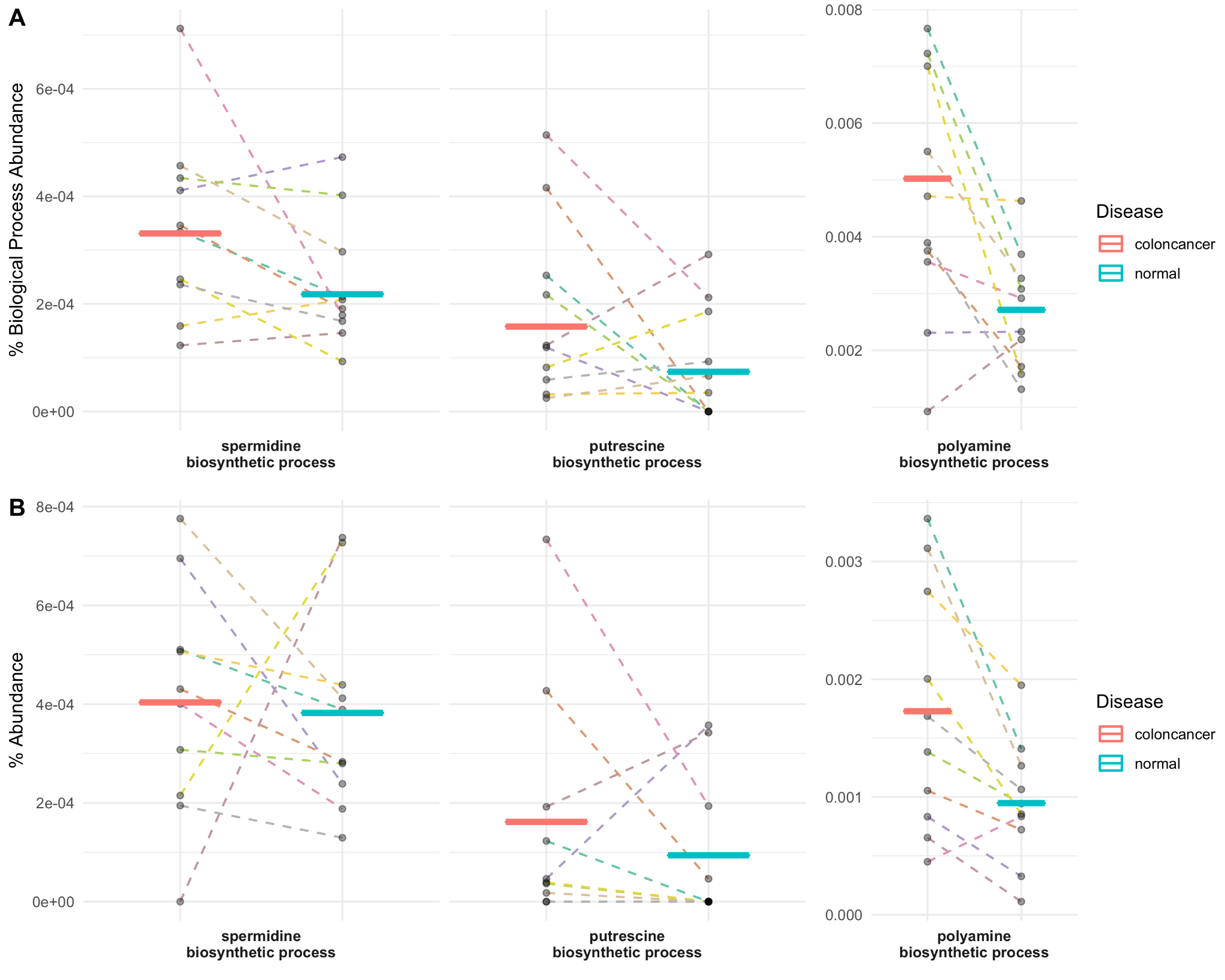 |
| --- |
| **Supplementary Figure S3. Polyamine biosynthetic process GO terms compared between CRC (red) and normal (blue) samples. A. As measured by MetaFunc.** Percent abundance of specific polyamine biosynthetic process GO terms among all biological process GOs in a sample/group. Values were calculated as described in section *2.3.2.4 Gene Ontology: Protein Annotation,* and output in MetaFunc tables or in the R Shiny application. These values were plotted, overlaying group means (horizontal lines) and individual values (data points). Line colors depict samples from a single patient. Lines connect paired CRC and normal samples. **B. As measured by HUMAnN2.** Percent abundance of specific polyamine biosynthetic process GO terms. HUMAnN2 outputted read counts as reads-per-kilobase (RPKs), which was then converted to counts per million (CPM) by dividing the RPK of a gene family by the total RPK in a sample. From CPM, percent abundance was obtained. |

##

##

##

## **Supplementary Tables**

| **Supplementary Table S1. Top 25 Gene Sets Enriched in CRC Samples from PRJNA413956 Dataset as Measured by Normalized Enrichment Scores.** | | | |
| --- | --- | --- | --- |
| **GO ID** | **GO Term** | **\|NES\|** | **p.adjust** |
| GO:0042254 | GO_RIBOSOME_BIOGENESIS | 2.54 | 0.0021 |
| GO:0000779 | GO_CONDENSED_CHROMOSOME_CENTROMERIC_REGION | 2.50 | 0.0021 |
| GO:0034660 | GO_NCRNA_METABOLIC_PROCESS | 2.49 | 0.0021 |
| GO:0000793 | GO_CONDENSED_CHROMOSOME | 2.47 | 0.0021 |
| GO:0006260 | GO_DNA_REPLICATION | 2.47 | 0.0021 |
| GO:0098687 | GO_CHROMOSOMAL_REGION | 2.47 | 0.0021 |
| GO:0140014 | GO_MITOTIC_NUCLEAR_DIVISION | 2.46 | 0.0021 |
| GO:0022613 | GO_RIBONUCLEOPROTEIN_COMPLEX_BIOGENESIS | 2.46 | 0.0021 |
| GO:0034470 | GO_NCRNA_PROCESSING | 2.46 | 0.0021 |
| GO:0016072 | GO_RRNA_METABOLIC_PROCESS | 2.45 | 0.0021 |
| GO:0006261 | GO_DNA_DEPENDENT_DNA_REPLICATION | 2.45 | 0.0021 |
| GO:0007059 | GO_CHROMOSOME_SEGREGATION | 2.45 | 0.0021 |
| GO:0000819 | GO_SISTER_CHROMATID_SEGREGATION | 2.45 | 0.0021 |
| GO:0000775 | GO_CHROMOSOME_CENTROMERIC_REGION | 2.44 | 0.0021 |
| GO:0000070 | GO_MITOTIC_SISTER_CHROMATID_SEGREGATION | 2.44 | 0.0021 |
| GO:0071103 | GO_DNA_CONFORMATION_CHANGE | 2.43 | 0.0021 |
| GO:0098813 | GO_NUCLEAR_CHROMOSOME_SEGREGATION | 2.39 | 0.0021 |
| GO:0000776 | GO_KINETOCHORE | 2.38 | 0.0021 |
| GO:0030684 | GO_PRERIBOSOME | 2.37 | 0.0021 |
| GO:0051983 | GO_REGULATION_OF_CHROMOSOME_SEGREGATION | 2.37 | 0.0021 |
| GO:0048285 | GO_ORGANELLE_FISSION | 2.37 | 0.0021 |
| GO:0006405 | GO_RNA_EXPORT_FROM_NUCLEUS | 2.36 | 0.0021 |
| GO:0000075 | GO_CELL_CYCLE_CHECKPOINT | 2.36 | 0.0021 |
| GO:0051783 | GO_REGULATION_OF_NUCLEAR_DIVISION | 2.35 | 0.0021 |
| GO:0034728 | GO_NUCLEOSOME_ORGANIZATION | 2.34 | 0.0021 |
| GO:0032200 | GO_TELOMERE_ORGANIZATION | 2.33 | 0.0021 |

| **Supplementary Table S2. Top 25 Gene Sets Enriched in CMS1 Dataset as Measured by Normalized Enrichment Scores against CMS2.** | | | |
| --- | --- | --- | --- |
| **GO ID** | **GO Term** | **\|NES\|** | **p.adjust** |
| GO:0034341 | GO_RESPONSE_TO_INTERFERON_GAMMA | 2.62 | 0.0022 |
| GO:0060333 | GO_INTERFERON_GAMMA_MEDIATED_SIGNALING_PATHWAY | 2.53 | 0.0022 |
| GO:0002250 | GO_ADAPTIVE_IMMUNE_RESPONSE | 2.49 | 0.0022 |
| GO:0007159 | GO_LEUKOCYTE_CELL_CELL_ADHESION | 2.40 | 0.0022 |
| GO:0051607 | GO_DEFENSE_RESPONSE_TO_VIRUS | 2.39 | 0.0022 |
| GO:0042110 | GO_T_CELL_ACTIVATION | 2.38 | 0.0022 |
| GO:0045088 | GO_REGULATION_OF_INNATE_IMMUNE_RESPONSE | 2.37 | 0.0022 |
| GO:0001909 | GO_LEUKOCYTE_MEDIATED_CYTOTOXICITY | 2.37 | 0.0022 |
| GO:0001906 | GO_CELL_KILLING | 2.36 | 0.0022 |
| GO:0034340 | GO_RESPONSE_TO_TYPE_I_INTERFERON | 2.36 | 0.0022 |
| GO:0031341 | GO_REGULATION_OF_CELL_KILLING | 2.36 | 0.0022 |
| GO:0050863 | GO_REGULATION_OF_T_CELL_ACTIVATION | 2.36 | 0.0022 |
| GO:0002449 | GO_LYMPHOCYTE_MEDIATED_IMMUNITY | 2.35 | 0.0022 |
| GO:0002228 | GO_NATURAL_KILLER_CELL_MEDIATED_IMMUNITY | 2.34 | 0.0022 |
| GO:1903039 | GO_POSITIVE_REGULATION_OF_LEUKOCYTE_CELL_CELL_ADHESION | 2.33 | 0.0022 |
| GO:0002703 | GO_REGULATION_OF_LEUKOCYTE_MEDIATED_IMMUNITY | 2.32 | 0.0022 |
| GO:0042098 | GO_T_CELL_PROLIFERATION | 2.32 | 0.0022 |
| GO:0002697 | GO_REGULATION_OF_IMMUNE_EFFECTOR_PROCESS | 2.32 | 0.0022 |
| GO:0032609 | GO_INTERFERON_GAMMA_PRODUCTION | 2.32 | 0.0022 |
| GO:0009615 | GO_RESPONSE_TO_VIRUS | 2.31 | 0.0022 |
| GO:0001818 | GO_NEGATIVE_REGULATION_OF_CYTOKINE_PRODUCTION | 2.30 | 0.0022 |
| GO:0098542 | GO_DEFENSE_RESPONSE_TO_OTHER_ORGANISM | 2.30 | 0.0022 |
| GO:0002237 | GO_RESPONSE_TO_MOLECULE_OF_BACTERIAL_ORIGIN | 2.30 | 0.0022 |
| GO:0050852 | GO_T_CELL_RECEPTOR_SIGNALING_PATHWAY | 2.30 | 0.0022 |
| GO:0001819 | GO_POSITIVE_REGULATION_OF_CYTOKINE_PRODUCTION | 2.30 | 0.0022 |

| **Supplementary Table S3. Top 25 Gene Sets Enriched in CMS1 Dataset as Measured by Normalized Enrichment Scores against CMS3.** | | | |
| --- | --- | --- | --- |
| **GO ID** | **GO Term** | **\|NES\|** | **p.adjust** |
| GO:0051607 | GO_DEFENSE_RESPONSE_TO_VIRUS | 2.38 | 0.0024 |
| GO:0034341 | GO_RESPONSE_TO_INTERFERON_GAMMA | 2.34 | 0.0024 |
| GO:0009615 | GO_RESPONSE_TO_VIRUS | 2.32 | 0.0024 |
| GO:0098542 | GO_DEFENSE_RESPONSE_TO_OTHER_ORGANISM | 2.28 | 0.0024 |
| GO:0071216 | GO_CELLULAR_RESPONSE_TO_BIOTIC_STIMULUS | 2.28 | 0.0024 |
| GO:0045088 | GO_REGULATION_OF_INNATE_IMMUNE_RESPONSE | 2.26 | 0.0024 |
| GO:0048002 | GO_ANTIGEN_PROCESSING_AND_PRESENTATION_OF_PEPTIDE_ANTIGEN | 2.25 | 0.0024 |
| GO:0034340 | GO_RESPONSE_TO_TYPE_I_INTERFERON | 2.25 | 0.0024 |
| GO:0030199 | GO_COLLAGEN_FIBRIL_ORGANIZATION | 2.24 | 0.0024 |
| GO:0032611 | GO_INTERLEUKIN_1_BETA_PRODUCTION | 2.23 | 0.0024 |
| GO:0019882 | GO_ANTIGEN_PROCESSING_AND_PRESENTATION | 2.22 | 0.0024 |
| GO:0042590 | GO_ANTIGEN_PROCESSING_AND_PRESENTATION_OF_EXOGENOUS_PEPTIDE_ ANTIGEN_VIA_MHC_CLASS_I | 2.22 | 0.0024 |
| GO:0032612 | GO_INTERLEUKIN_1_PRODUCTION | 2.21 | 0.0024 |
| GO:0060333 | GO_INTERFERON_GAMMA_MEDIATED_SIGNALING_PATHWAY | 2.20 | 0.0024 |
| GO:0002474 | GO_ANTIGEN_PROCESSING_AND_PRESENTATION_OF_PEPTIDE_ANTIGEN_ VIA_MHC_CLASS_I | 2.18 | 0.0024 |
| GO:0005201 | GO_EXTRACELLULAR_MATRIX_STRUCTURAL_CONSTITUENT | 2.18 | 0.0024 |
| GO:0043062 | GO_EXTRACELLULAR_STRUCTURE_ORGANIZATION | 2.18 | 0.0024 |
| GO:0001819 | GO_POSITIVE_REGULATION_OF_CYTOKINE_PRODUCTION | 2.18 | 0.0024 |
| GO:0050663 | GO_CYTOKINE_SECRETION | 2.18 | 0.0024 |
| GO:0005581 | GO_COLLAGEN_TRIMER | 2.17 | 0.0024 |
| GO:0032635 | GO_INTERLEUKIN_6_PRODUCTION | 2.17 | 0.0024 |
| GO:0070555 | GO_RESPONSE_TO_INTERLEUKIN_1 | 2.17 | 0.0024 |
| GO:0071706 | GO_TUMOR_NECROSIS_FACTOR_SUPERFAMILY_CYTOKINE_PRODUCTION | 2.15 | 0.0024 |
| GO:0071887 | GO_LEUKOCYTE_APOPTOTIC_PROCESS | 2.15 | 0.0024 |
| GO:0031349 | GO_POSITIVE_REGULATION_OF_DEFENSE_RESPONSE | 2.12 | 0.0024 |

| **Supplementary Table S4. Top 25 Gene Sets Enriched in CMS2 Dataset as Measured by Normalized Enrichment Scores against CMS1.** | | | |
| --- | --- | --- | --- |
| **GO ID** | **GO Term** | **\|NES\|** | **p.adjust** |
| GO:0042273 | GO_RIBOSOMAL_LARGE_SUBUNIT_BIOGENESIS | 2.10 | 0.0042 |
| GO:0140053 | GO_MITOCHONDRIAL_GENE_EXPRESSION | 2.04 | 0.0056 |
| GO:0032543 | GO_MITOCHONDRIAL_TRANSLATION | 1.97 | 0.0052 |
| GO:0042788 | GO_POLYSOMAL_RIBOSOME | 1.93 | 0.0035 |
| GO:0008033 | GO_TRNA_PROCESSING | 1.93 | 0.0052 |
| GO:0044391 | GO_RIBOSOMAL_SUBUNIT | 1.92 | 0.006 |
| GO:0042254 | GO_RIBOSOME_BIOGENESIS | 1.92 | 0.0079 |
| GO:0000462 | GO_MATURATION_OF_SSU_RRNA_FROM_TRICISTRONIC_RRNA_TRANSCRIPT_  SSU_RRNA_5_8S_RRNA_LSU_RRNA | 1.92 | 0.0037 |
| GO:0030490 | GO_MATURATION_OF_SSU_RRNA | 1.90 | 0.0063 |
| GO:0034660 | GO_NCRNA_METABOLIC_PROCESS | 1.90 | 0.0116 |
| GO:0006400 | GO_TRNA_MODIFICATION | 1.90 | 0.0044 |
| GO:0016072 | GO_RRNA_METABOLIC_PROCESS | 1.90 | 0.0063 |
| GO:0006399 | GO_TRNA_METABOLIC_PROCESS | 1.89 | 0.0059 |
| GO:0000184 | GO_NUCLEAR_TRANSCRIBED_MRNA_CATABOLIC_PROCESS_NONSENSE_  MEDIATED_DECAY | 1.88 | 0.0051 |
| GO:0006270 | GO_DNA_REPLICATION_INITIATION | 1.88 | 0.0060 |
| GO:0003735 | GO_STRUCTURAL_CONSTITUENT_OF_RIBOSOME | 1.88 | 0.0056 |
| GO:0030684 | GO_PRERIBOSOME | 1.88 | 0.0042 |
| GO:0042274 | GO_RIBOSOMAL_SMALL_SUBUNIT_BIOGENESIS | 1.88 | 0.004 |
| GO:0070129 | GO_REGULATION_OF_MITOCHONDRIAL_TRANSLATION | 1.87 | 0.0097 |
| GO:0034470 | GO_NCRNA_PROCESSING | 1.85 | 0.0097 |
| GO:0005736 | GO_RNA_POLYMERASE_I_COMPLEX | 1.84 | 0.0087 |
| GO:0001510 | GO_RNA_METHYLATION | 1.84 | 0.0043 |
| GO:0098798 | GO_MITOCHONDRIAL_PROTEIN_COMPLEX | 1.84 | 0.0073 |
| GO:0015934 | GO_LARGE_RIBOSOMAL_SUBUNIT | 1.82 | 0.005 |
| GO:0022626 | GO_CYTOSOLIC_RIBOSOME | 1.81 | 0.005 |

| **Supplementary Table S5. Top 25 Gene Sets Enriched in CMS2 Dataset as Measured by Normalized Enrichment Scores against CMS3.** | | | |
| --- | --- | --- | --- |
| **GO ID** | **GO Term** | **\|NES\|** | **p.adjust** |
| GO:0034660 | GO_NCRNA_METABOLIC_PROCESS | 2.48 | 0.0029 |
| GO:0042254 | GO_RIBOSOME_BIOGENESIS | 2.46 | 0.0029 |
| GO:0034470 | GO_NCRNA_PROCESSING | 2.45 | 0.0029 |
| GO:0022613 | GO_RIBONUCLEOPROTEIN_COMPLEX_BIOGENESIS | 2.44 | 0.0029 |
| GO:0016072 | GO_RRNA_METABOLIC_PROCESS | 2.43 | 0.0029 |
| GO:0030684 | GO_PRERIBOSOME | 2.41 | 0.0029 |
| GO:0006270 | GO_DNA_REPLICATION_INITIATION | 2.40 | 0.0029 |
| GO:0032543 | GO_MITOCHONDRIAL_TRANSLATION | 2.31 | 0.0029 |
| GO:0006399 | GO_TRNA_METABOLIC_PROCESS | 2.29 | 0.0029 |
| GO:0032040 | GO_SMALL_SUBUNIT_PROCESSOME | 2.28 | 0.0029 |
| GO:0140053 | GO_MITOCHONDRIAL_GENE_EXPRESSION | 2.28 | 0.0029 |
| GO:0042273 | GO_RIBOSOMAL_LARGE_SUBUNIT_BIOGENESIS | 2.21 | 0.0029 |
| GO:0008033 | GO_TRNA_PROCESSING | 2.20 | 0.0029 |
| GO:0006415 | GO_TRANSLATIONAL_TERMINATION | 2.16 | 0.0029 |
| GO:0007143 | GO_FEMALE_MEIOTIC_NUCLEAR_DIVISION | 2.14 | 0.0029 |
| GO:0120114 | GO_SM_LIKE_PROTEIN_FAMILY_COMPLEX | 2.11 | 0.0029 |
| GO:0000313 | GO_ORGANELLAR_RIBOSOME | 2.09 | 0.0029 |
| GO:0044391 | GO_RIBOSOMAL_SUBUNIT | 2.08 | 0.0029 |
| GO:0003688 | GO_DNA_REPLICATION_ORIGIN_BINDING | 2.08 | 0.0029 |
| GO:0070126 | GO_MITOCHONDRIAL_TRANSLATIONAL_TERMINATION | 2.08 | 0.0029 |
| GO:0140101 | GO_CATALYTIC_ACTIVITY_ACTING_ON_A_TRNA | 2.08 | 0.0029 |
| GO:0071826 | GO_RIBONUCLEOPROTEIN_COMPLEX_SUBUNIT_ORGANIZATION | 2.02 | 0.0029 |
| GO:0000375 | GO_RNA_SPLICING_VIA_TRANSESTERIFICATION_REACTIONS | 2.02 | 0.0029 |
| GO:0015934 | GO_LARGE_RIBOSOMAL_SUBUNIT | 2.02 | 0.0029 |
| GO:0000184 | GO_NUCLEAR_TRANSCRIBED_MRNA_CATABOLIC_PROCESS_NONSENSE_  MEDIATED_DECAY | 2.02 | 0.0029 |

| **Supplementary Table S6. Top 25 Gene Sets Enriched in CMS3 Dataset as Measured by Normalized Enrichment Scores against CMS1.** | | | |
| --- | --- | --- | --- |
| **GO ID** | **GO Term** | **\|NES\|** | **p.adjust** |
| GO:0009812 | GO_FLAVONOID_METABOLIC_PROCESS | 2.22 | 0.0032 |
| GO:0072329 | GO_MONOCARBOXYLIC_ACID_CATABOLIC_PROCESS | 2.22 | 0.0037 |
| GO:0009062 | GO_FATTY_ACID_CATABOLIC_PROCESS | 2.19 | 0.0037 |
| GO:0006805 | GO_XENOBIOTIC_METABOLIC_PROCESS | 2.17 | 0.0037 |
| GO:0034440 | GO_LIPID_OXIDATION | 2.16 | 0.0037 |
| GO:0006635 | GO_FATTY_ACID_BETA_OXIDATION | 2.14 | 0.0035 |
| GO:0003707 | GO_STEROID_HORMONE_RECEPTOR_ACTIVITY | 2.11 | 0.0034 |
| GO:0006631 | GO_FATTY_ACID_METABOLIC_PROCESS | 2.07 | 0.0047 |
| GO:0044242 | GO_CELLULAR_LIPID_CATABOLIC_PROCESS | 2.06 | 0.0043 |
| GO:0006063 | GO_URONIC_ACID_METABOLIC_PROCESS | 2.04 | 0.0032 |
| GO:0034308 | GO_PRIMARY_ALCOHOL_METABOLIC_PROCESS | 2.04 | 0.0035 |
| GO:0016408 | GO_C_ACYLTRANSFERASE_ACTIVITY | 1.99 | 0.0032 |
| GO:0005903 | GO_BRUSH_BORDER | 1.99 | 0.0037 |
| GO:0045277 | GO_RESPIRATORY_CHAIN_COMPLEX_IV | 1.99 | 0.0032 |
| GO:0001972 | GO_RETINOIC_ACID_BINDING | 1.93 | 0.0064 |
| GO:0016614 | GO_OXIDOREDUCTASE_ACTIVITY_ACTING_ON_CH_OH_GROUP_OF_DONORS | 1.92 | 0.0037 |
| GO:0034754 | GO_CELLULAR_HORMONE_METABOLIC_PROCESS | 1.92 | 0.0036 |
| GO:0032787 | GO_MONOCARBOXYLIC_ACID_METABOLIC_PROCESS | 1.91 | 0.0060 |
| GO:0071280 | GO_CELLULAR_RESPONSE_TO_COPPER_ION | 1.91 | 0.0081 |
| GO:0004879 | GO_NUCLEAR_RECEPTOR_ACTIVITY | 1.90 | 0.0052 |
| GO:0016042 | GO_LIPID_CATABOLIC_PROCESS | 1.90 | 0.0050 |
| GO:0033559 | GO_UNSATURATED_FATTY_ACID_METABOLIC_PROCESS | 1.90 | 0.0035 |
| GO:0015701 | GO_BICARBONATE_TRANSPORT | 1.89 | 0.0068 |
| GO:0033293 | GO_MONOCARBOXYLIC_ACID_BINDING | 1.89 | 0.0055 |
| GO:0033540 | GO_FATTY_ACID_BETA_OXIDATION_USING_ACYL_COA_OXIDASE | 1.88 | 0.0093 |

| **Supplementary Table S7. Top 25 Gene Sets Enriched in CMS3 Dataset as Measured by Normalized Enrichment Scores against CMS2.** | | | |
| --- | --- | --- | --- |
| **GO ID** | **GO Term** | **\|NES\|** | **p.adjust** |
| GO:0006063 | GO_URONIC_ACID_METABOLIC_PROCESS | 2.09 | 0.0029 |
| GO:0071294 | GO_CELLULAR_RESPONSE_TO_ZINC_ION | 2.07 | 0.0029 |
| GO:0015020 | GO_GLUCURONOSYLTRANSFERASE_ACTIVITY | 2.06 | 0.0029 |
| GO:0009812 | GO_FLAVONOID_METABOLIC_PROCESS | 2.05 | 0.0029 |
| GO:0071276 | GO_CELLULAR_RESPONSE_TO_CADMIUM_ION | 1.98 | 0.0029 |
| GO:0010043 | GO_RESPONSE_TO_ZINC_ION | 1.96 | 0.0029 |
| GO:0006805 | GO_XENOBIOTIC_METABOLIC_PROCESS | 1.96 | 0.0029 |
| GO:0034754 | GO_CELLULAR_HORMONE_METABOLIC_PROCESS | 1.96 | 0.0029 |
| GO:0004745 | GO_RETINOL_DEHYDROGENASE_ACTIVITY | 1.94 | 0.0029 |
| GO:0005496 | GO_STEROID_BINDING | 1.92 | 0.0029 |
| GO:0030258 | GO_LIPID_MODIFICATION | 1.91 | 0.0029 |
| GO:0016042 | GO_LIPID_CATABOLIC_PROCESS | 1.91 | 0.0029 |
| GO:0006635 | GO_FATTY_ACID_BETA_OXIDATION | 1.91 | 0.0029 |
| GO:0034440 | GO_LIPID_OXIDATION | 1.89 | 0.0029 |
| GO:0006654 | GO_PHOSPHATIDIC_ACID_BIOSYNTHETIC_PROCESS | 1.87 | 0.0029 |
| GO:0007586 | GO_DIGESTION | 1.86 | 0.0029 |
| GO:0006066 | GO_ALCOHOL_METABOLIC_PROCESS | 1.85 | 0.0029 |
| GO:0042572 | GO_RETINOL_METABOLIC_PROCESS | 1.83 | 0.0047 |
| GO:0001972 | GO_RETINOIC_ACID_BINDING | 1.82 | 0.0047 |
| GO:0016408 | GO_C_ACYLTRANSFERASE_ACTIVITY | 1.81 | 0.0078 |
| GO:0032411 | GO_POSITIVE_REGULATION_OF_TRANSPORTER_ACTIVITY | 1.81 | 0.0029 |
| GO:0005319 | GO_LIPID_TRANSPORTER_ACTIVITY | 1.81 | 0.0029 |
| GO:0044242 | GO_CELLULAR_LIPID_CATABOLIC_PROCESS | 1.80 | 0.0029 |
| GO:0008028 | GO_MONOCARBOXYLIC_ACID_TRANSMEMBRANE_TRANSPORTER_ACTIVITY | 1.79 | 0.0062 |
| GO:0042445 | GO_HORMONE_METABOLIC_PROCESS | 1.79 | 0.0029 |
